# Independent associations of anti-cyclic citrullinated peptide antibodies and rheumatoid factor with radiographic severity of rheumatoid arthritis

**DOI:** 10.1186/ar2017

**Published:** 2006-07-20

**Authors:** Devesh Mewar, Annabel Coote, David J Moore, Ioanna Marinou, Jodie Keyworth, Marion C Dickson, Doug S Montgomery, Michael H Binks, Anthony G Wilson

**Affiliations:** 1 Division of Genomic Medicine, University of Sheffield, Sheffield, UK; 2 GlaxoSmithKline R&D, Stevenage, UK

## Abstract

Several recent publications have established a strong association between anti-cyclic citrullinated peptide antibody (anti-CCP)-positive rheumatoid arthritis (RA) and carriage of shared epitope (SE) alleles. Although anti-CCP have also been associated with more severe RA, the issue of whether this is independent of rheumatoid factor (RF) has not been addressed. To identify associations between RF, anti-CCP, SE status and radiological damage, we studied a large cross-sectional cohort with longstanding RA. Individuals (*n *= 872) enrolled in the study all fulfilled the American College of Rheumatology criteria for RA, had a minimum disease duration of 3 years, and at least one definite radiographic erosion was present in hands or feet. Radiographs were scored blind at study entry by a single musculoskeletal radiologist using a modified Larsen's score. Anti-CCP and RF levels were determined using enzyme-linked immunosorbent assay, and DRB1 typing was performed using polymerase chain reaction based methodology. Both anti-CCP and RF levels were strongly associated with radiographic severity (*P *< 0.0001). In subgroups stratified for both anti-CCP and RF status, evidence of independent associations of both antibodies with radiographic outcome was found (*P *< 0.0001). An association of SE alleles with radiographic severity was present only in RF-negative individuals. Anti-CCP positivity was associated with SE status with evidence of a gene-dose effect, most markedly in RF-negative individuals (*P *< 0.01). Anti-CCP and RF status are independent severity factors for RA, with SE alleles playing at most a secondary role. Our data support the view that previously described associations between SE and radiological severity, especially in RF-negative patients, may be indirect and due to an association with anti-CCP.

## Introduction

Antibodies to cyclic citrullinated peptides (anti-CCP) exhibit high specificity for rheumatoid arthritis (RA) [1]. Recent studies demonstrated that shared epitope (SE) alleles are strongly associated with anti-CCP-positive but not anti-CCP-negative RA [2], and indeed are more strongly associated with anti-CCP than with RA itself [3,4]. These findings lend strong support to the concept of anti-CCP-positive RA as a distinct entity [2]. Furthermore, anti-CCP has been shown to influence radiographic progression in prospective studies, with some evidence of an interaction with SE alleles [2,5]. However, the presence of anti-CCP is associated with the presence of rheumatoid factor (RF) [3], which also is an established severity factor in RA in prospective studies of progression [6] as well as longstanding disease [7].

Whether associations of anti-CCP with disease severity are independent of RF remains unclear. The influence of SE alleles on disease severity appears to vary among populations, with most studies suggesting an association with erosivity [8]. Several studies have suggested that the association of SE alleles with radiographic outcome is relevant only in RF-negative individuals [8-10]. Because carriage of SE alleles is associated with anti-CCP but not RF, it was recently suggested [3] that effects of SE alleles on disease severity may be indirect and secondary to an association with anti-CCP. In the present study we determined associations between radiographic outcome in longstanding disease (as assessed by modified Larsen's score) and RF, anti-CCP and SE alleles. We report independent associations of RF and anti-CCP with radiographic severity of disease, suggesting that both of these factors may have important influence on pathways that lead to joint damage. We concur with previous studies suggesting that SE alleles are associated with radiographic outcome only in RF-negative individuals, and confirm a strong association between anti-CCP status and SE alleles, with evidence of a gene-dose effect. This association is most striking in RF-negative individuals, supporting the hypothesis that the association of SE alleles with disease severity in RA may act via anti-CCP.

## Materials and methods

### Participants

Patients with established RA attending outpatient clinics at the Royal Hallamshire Hospital, Sheffield, UK were enrolled in the study between 1999 and 2006. Research ethics committee approval was obtained for the study (SSREC protocol number 02/186) and all participants gave informed consent. All subjects were white Caucasian, fulfilled the American College of Rheumatology criteria for RA [11], had a minimum disease duration of 3 years, and had at least one definite radiographic erosion in hands or feet. Radiographs of hands and feet were scored blind at study entry by a single musculoskeletal radiologist (DJM) using a modification to Larsen's score [12]. To check whether scoring was consistent, 10% of films were selected at random and returned for repeat blinded analysis. A weighted kappa score was calculated to quantify the intra-observer variation in the modified Larsen scoring. The weighted kappa score was 0.83, indicating very good agreement between the initial and repeat scores.

### Antibody measurement and genotyping

Anti-CCP levels were measured using the DIASTAT™ anti-CCP ELISA (Axis-Shield, Cambs, UK). The semiquantitative protocol was used as recommended by the manufacturer and a cut-off of 5.5 units/ml was established based on mean plus three standard deviations of values obtained from 100 age-matched control individuals. RF was measured using a nephelometric method on the Dade Behring BN2 nephelometer. Genotyping for DRB1 alleles was performed using polymerase chain reaction based methods [13]. The following DRB1 alleles were classified as SE alleles: *0101, *0102, *0104, *0401, *0404, *0405, *0408, *0409, *0410, *0413, *0416, *0419, *0421 and *1001.

### Statistical analyses

Quantitative variables were compared between groups by Student's t-test or by one-way analysis of variance, as appropriate. Proportions of anti-CCP-positive individuals were compared between subgroups by χ^2 ^tests with two degrees of freedom and are described as odds ratios with 95% confidence limits derived using Miettinen's test-based approximation.

## Results

### Characteristics of the study population

A total of 872 individuals were included in the cohort. The mean disease duration was 18 years (range 3–65 years). Mean age at onset was 41 years (range 17–80 years) and mean age at assessment was 59 years. The proportion of females in the cohort was 73%. The majority of patients (93.7%) had been treated with disease-modifying agents. Neither disease duration nor the proportion of DMARD (disease-modifying antirheumatic drug)-naïve individuals was significantly different between the subgroups analyzed in Tables 1, 2, 3 below (data not shown).

### Association among radiographic outcome, RF and anti-CCP status

In our cohort both anti-CCP and RF were strongly associated with radiographic severity, as measured using Larsen's score (*P *< 0.0001), as was reported previously (Table 1). Additionally, in subgroups stratified for both anti-CCP and RF status we found evidence of independent associations of both RF and anti-CCP with radiographic outcome. Those with both RF and anti-CCP expressed the most severe disease (mean Larsen's score 49.6); those with RF but not anti-CCP, or without RF but with anti-CCP were intermediate in terms of severity; and double-negative patients had the mildest disease (*P *< 0.0001, analysis of variance).

### Association between SE copy number and radiographic outcome in patients stratified for RF and anti-CCP

We next examined the potential influence of SE alleles on radiographic outcome in our cohort by determining associations between copy number of SE alleles and Larsen's score in patients stratified for both RF and anti-CCP status. A weak association of SE alleles with radiographic severity was found only in RF-negative patients, with evidence of a gene-dose effect (Table 2). Although a trend toward an association between SE copy number and radiographic outcome was seen in the cohort as a whole (Table 2), this was not statistically significant. Notably such a trend was not apparent in anti-CCP-positive or anti-CCP-negative subgroups.

### SE copy number is associated with anti-CCP status

Because both anti-CCP positivity and levels have been associated with SE status, we were interested in the magnitude of this association in our cohort. We also found strong associations between carriage of SE alleles and anti-CCP status, with evidence of a gene-dose effect (Table 3). This was statistically significant both in the cohort as a whole as well as in RF-positive and RF-negative subgroups, but the magnitude of this effect was maximal in RF-negative patients, with an odds ratio of 6.16 for anti-CCP positivity in individuals with two SE alleles compared with none. Conversely, the magnitude of this effect was smallest in the RF-positive patients. We found no association between SE copy number and RF status in a similar analysis (data not shown).

## Discussion

Clarification of the relationship between DRB1 alleles, RF, anti-CCP and disease severity remains a matter of considerable importance both clinically (prediction of disease phenotype and the design of clinical trials/studies) and because of the pathogenetic insights that may result. Recent studies have highlighted the association between anti-CCP and radiographic progression in early RA. However, because RF and anti-CCP coexist in the majority of individuals with RA, few studies examining the influence of anti-CCP on disease severity have been sufficiently powered to stratify for RF status. In one such study of radiographic progression [14], the presence of RF independently of anti-CCP did not appear to influence progression of early disease. We found that anti-CCP antibodies are strongly associated with radiographic outcome in a cross-sectional cohort with established disease. In our study the presence of RF alone and anti-CCP alone were associated with similar radiographic outcomes, with patients without either antibody clearly accumulating less damage. This finding suggests that both RF and anti-CCP are independent severity factors for RA.

The association between SE alleles and radiographic outcome in RA has been extensively debated [15]. The situation is considered to be complex, with ethnic background and other factors playing significant roles. Most recent analyses [8] suggested that the primary association of SE alleles is with the development of erosions, especially in RF-negative individuals [9,10]. The influence of SE alleles on severity of radiographic damage is less clear, but again there is evidence of an association in RF-negative individuals [7]. We also found a weak association between SE copy number and severity of radiographic damage in RF-negative patients only. In our cohort anti-CCP status was strongly associated with the SE, as has been reported previously, but a clear gene-dose effect was also evident. The magnitude of this effect was most striking in RF-negative patients, which supports the view that the association of SE with radiographic severity may be indirect and due to an association with anti-CCP. Because the proportion of RF-positive/SE negative individuals who were anti-CCP positive was still very high, at 83%, this may explain why no association between SE and radiographic severity was seen in this group.

The large sample size of our study is a strength and allows robust subgroup analyses; however, the cross-sectional design and selection of patients with longstanding and more severe (erosive) disease do raise the possibility of selection bias, and therefore the findings may not be generalizable to populations with milder disease. Nevertheless, we believe that our findings have some direct practical implications. They suggest that determinations of RF and anti-CCP have at least equivalent value in predicting the severity of radiographic damage in RA, with typing of DRB1 alleles being likely to provide little added information. They also support the concept that anti-CCP positive disease is a distinct pathogenetic entity associated with SE alleles, and that different pathways are responsible for driving the production of anti-CCP and RF.

## Conclusion

We demonstrated that radiographic severity of RA is associated independently with both RF and anti-CCP. In addition, the finding that SE alleles are more strongly associated with anti-CCP in RF-negative individuals provides an explanation for the previously reported observation that SE alleles are only associated with radiographic severity in RF-negative individuals. These findings support the hypothesis that the association of SE alleles with RA severity may be secondary to anti-CCP positivity.

## Abbreviations

anti-CCP = anti-cyclic citrullinated peptide antibody; RA = rheumatoid arthritis; RF = rheumatoid factors; SE = shared epitope.

## Competing interests

The authors declare that they have no competing interests.

## Authors' contributions

DM, MCD, DSM, MHB and AGW designed the study. AC, DJM, IM and JK acquired the data. DM performed data analysis and all of the authors were involved in the interpretation of the data. All of the authors read and approved the final manuscript.

## Figures and Tables

**Table 1:** Radiographic damage in patients stratified for RF and anti-CCP status

Antibody status	*n*	Larsen's score (mean ± SEM)	*P*
RF positive	552	49.1 ± 1.7	<0.0001^a^
RF negative	268	33.0 ± 1.9	
			
Anti-CCP positive	672	46.9 ± 1.5	<0.0001^a^
Anti-CCP negative	159	31.3 ± 2.4	
			
RF positive/CCP positive	493	49.6 ± 1.8	<0.0001^b^
RF positive/CCP negative	39	37.6 ± 5.2	
RF negative/CCP positive	143	37.2 ± 2.7	
RF negative/CCP negative	109	28.0 ± 2.8	

Shown is an analysis of radiographic damage, as assessed by modified Larsen's score, in patients stratified for RF and anti-CCP status. ^a^t-test. ^b^Comparison of four groups by analysis of variance. anti-CCP, anti-cyclic citrullinated peptide antibody; RF, rheumatoid factor; SEM, standard error of the mean.

**Table 2:** Radiographic damage among patients with two, one, or no copies of SE alleles stratified according to RF and anti-CCP status

Antibody status	Number of SE alleles	*n*	Larsen's score (mean ± SEM)	*P *(SE2 versus SE1 versus SE0)
All	2	266	46.5 ± 2.3	NS
	1	393	43.6 ± 1.9	
	0	168	39.2 ± 2.6	
				
RF positive	2	177	49.5 ± 2.9	NS
	1	253	49.4 ± 2.5	
	0	94	47.0 ± 3.7	
				
RF negative	2	71	39.0 ± 4.3	0.0458^a^
	1	120	32.3 ± 2.7	
	0	65	25.9 ± 3.1	
				
CCP positive	2	226	49.1 ± 2.5	NS
	1	312	45.7 ± 2.1	
	0	98	44.3 ± 3.4	
				
CCP negative	2	30	32.0 ± 5.9	NS
	1	67	32.3 ± 3.7	
	0	58	29.2 ± 3.9	

Shown is an analysis of radiographic damage among patients with 2 (SE2), 1 (SE1) or no (SE0) copies of SE alleles stratified according to RF and anti-CCP (shortened to CCP) status. ^a^By analysis of variance. anti-CCP, anti-cyclic citrullinated peptide antibody; NS, not significant; RF, rheumatoid factor; SEM, standard error of the mean.

**Table 3:** Association between SE alleles and anti-CCP status

RF status	Number of SE alleles	Proportion anti-CCP positive	OR (95% CI)	*P*
All	2	214/241	4.94 (2.94–8.22)	<0.001
	1	307/373	2.90 (1.89–4.43)	
	0	98/159	1	
				
RF positive	2	162/170	4.15 (1.79–9.58)	<0.01
	1	236/253	2.85 (1.41–5.77)	
	0	78/94	1	
				
RF negative	2	52/71	6.16 (3.00–12.6)	<0.001
	1	71/120	3.26 (1.74–6.11)	
	0	20/65	1	

Absolute numbers of anti-CCP-positive patients in each subgroup are given. OR with 95% CI are shown for anti-CCP positivity associated with carriage of two or one SE allele versus no SE alleles for RF positive, RF negative and combined subgroups. *P *values are for χ^2 ^tests with two degrees of freedom comparing the proportion of anti-CCP positive patients in subgroups carrying two, one, or no SE alleles. anti-CCP, anti-cyclic citrullinated peptide antibody; CI, confidence interval; NS, not significant; OR, odds ratio; RF, rheumatoid factor; SEM, standard error of the mean.
